# A Facile Synthesis of TiO_2_–α-Ga_2_O_3_-Based Self-Powered Broad-Band UVC/UVA Photodetector and Optical Communication Study

**DOI:** 10.3390/ma17164103

**Published:** 2024-08-19

**Authors:** Wenxing Zhang, Anqi Xu, Xin Zhou, Dan Zhang, Honglin Li

**Affiliations:** Chongqing Key Laboratory of Photo-Electric Functional Materials, College of Physics and Electronic Engineering, Chongqing Normal University, Chongqing 401331, China; zwxhmhxz2003@163.com (W.Z.); axxxlash@gmail.com (X.Z.); levo_7@163.com (D.Z.)

**Keywords:** solar blind, Ga_2_O_3_, ultraviolet photodetector, communication

## Abstract

Traditional optical communication systems rely on single narrow-band PDs, which can expose confidential information and data to potential eavesdropping in free space. With advancements in technology, even optical communication in the UV spectrum, invisible to the sun, faces risks of interception. Consequently, broad-band PDs that combine optical encryption with algorithmic encryption hold significant promise for secure and reliable communication. This study presents a photodetector based on TiO_2_–α-Ga_2_O_3_ heterostructures, prepared via direct oxidation and hydrothermal reaction, demonstrating self-powered UVC/UVA broad-band detection capabilities. The PD exhibits response peaks at approximately 250 and 320 nm, with R of 42.16 and 59.88 mA/W and D* of 8.21 × 10^13^ and 9.56 × 10^13^ Jones, respectively. Leveraging the superior optical response characteristics of UVC and UVA wavelengths, this device has been employed to develop a communication system designed for data transmission. The proposed system features two independent channels: one for data transmission using UVC and another for key distribution using UVA. Secure communication is ensured through specialized encryption algorithms. In summary, this work offers a straightforward, cost-effective, and practical method for fabricating self-powered UVC/UVA broad-band PDs. This PD provides new insights into the development of multi-purpose, multi-band secure optical communication devices and holds promise for integration into multifunctional optoelectronic systems in the future.

## 1. Introduction

UV PDs are essential components in advanced communication, ozone monitoring, fire detection, etc. [1,2]. UV radiation is generally classified into three bands: UVA, UVB, and UVC (400–315–280–100 nm) [3]. Among these bands, UVA and UVC radiation have notable impacts on our daily lives, encompassing both positive and negative effects. UVC radiation is extensively utilized in various military and civilian applications, including flame detection, environmental monitoring, and missile warning systems [4,5,6]. UVA PDs can be employed for ore identification, stage decoration, banknote inspection, and wearable devices to record the UV index, which helps prevent the harmful effects of sun exposure on the skin. Conversely, excessive exposure to UVC/UVA radiation can penetrate the dermis of the skin, damaging elastic and collagen fibers and even triggering skin cancer [7,8,9]. Therefore, precise detection of UVC and UVA radiation is critically important. Due to the increasing need for more adaptable UV photodetectors, substantial efforts have been directed toward achieving multispectral photodetection. This has been accomplished through ongoing innovations in advanced semiconductor materials, growth techniques, and device architectures [10,11]. Silicon-based detectors are relatively mature, while they often necessitate high-transmittance filters and phosphor materials to block low-energy photons and enhance efficiency, thereby increasing manufacturing and operational costs [12]. Wu et al. developed a dual-band photodetector that utilizes N-films produced from an AlGaN layer through a thermal oxidation process [13]. This LED achieves two distinct response peaks at approximately 200 and 305 nm. By varying the bias voltage from 10 to 25 V, the photodetector operates in UVB-dominant, UVC/UVB broad-band, and UVC-dominant detection modes, showcasing a tunable dual-band optical detection capability. Developing high-performance multi-band PDs remains a significant challenge, particularly those capable of broad-band detection in the UVC and UVA regions, which have been scarcely documented. In this study, a TiO_2_–α-Ga_2_O_3_ heterostructure was fabricated using direct oxidation and hydrothermal reaction methods, resulting in a photodetector with self-powered UVC/UVA broad-band detection capability. In contrast to previous designs requiring additional external voltage, this approach achieves self-powering, simplifying the experimental setup and minimizing interference from external electric fields. This offers a straightforward and cost-effective method for producing self-powered UVC/UVA broad-band photodetectors and holds promise for advancing research into versatile, flexible multi-band optical communication devices. Wide bandgap semiconductor materials can achieve UV detection without the need for special treatments of materials or devices for their inherent bandgap advantages [14,15,16]. With advancements in material growth technology and device fabrication capabilities, acquiring wide bandgap semiconductor materials has become easier, leading to significant progress in wide bandgap semiconductor UV detectors. In this context, AlGaN and MgZnO have attracted significant attention. The bandgap of AlGaN can be adjusted within the range of 3.4–6.2 eV, allowing AlGaN-based PDs to detect deep ultraviolet (DUV) varying from 200 to 280 nm, UVB, and UVA light [17,18,19]. However, the epitaxial growth of high-Al-content AlGaN with high crystalline quality remains a challenge due to structural defects caused by lattice and thermal expansion mismatches in the epitaxial structure [20]. Meanwhile, Ga_2_O_3_ has emerged as one of the most promising candidates for DUV PDs because of its intrinsically ultrawide bandgap, which has a cut-off wavelength below 280 nm. Specifically, Qian et al. achieved an R of 216 A/W and a D* of 4.22 × 10^15^ Jones for Ga_2_O_3_-based DUV detectors through the employment of localized surface plasmon resonance via an Al@Al_2_O_3_ core–shell nanostructure array [21]. Liu et al. achieved ultra-high DUV photoresponse performance by constructing an asymmetric barrier Ga_2_O_3_-based Schottky junction, enabling the detector to operate stably in a passive state (0 V bias) [22]. This resulted in an R of 0.73 mA/W and a D* of 3.35 × 10^10^ Jones.

Regarding the significance and applications of broad-band detection, single narrow-band PDs are inadequate for meeting the demands of high-precision target detection and information recognition [23,24,25]. Broad-band and multi-band PDs, however, can leverage the photoresponse characteristics across different bands to achieve mutual verification and compensation of information, thus enhancing the accuracy, precision, and safety of data. Broad-band PDs, including visible near-infrared PDs [26], UV near-infrared PDs [27], and UV-visible PDs [28], are extensively used in applications such as flame detection, memory storage, and secure communication. PDs that achieve broad-band or even multi-band detection within a single device offer advantages such as simplified structure and reduced interference. For instance, He et al. developed UV-infrared PDs based on β-Ga_2_O_3_/BP heterojunctions [29], while Wang et al. fabricated PDs based on MoS_2_/Si heterojunctions, demonstrating photoresponse from the visible to the infrared region [30].

For most electronic and optoelectronic devices, heterojunctions bring numerous novel physical phenomena and improvements in the device due to differing bandgaps and discontinuous energy bands [31,32]. The correct and rational selection of materials plays a vital role in improving heterojunction devices. Notably, TiO_2_ is well suited for constructing UV PDs due to its strong absorption in the UV region, simple preparation process, and ease of crystal phase control [33]. For instance, Reddy et al. synthesized TiO_2_–α-Ga_2_O_3_ composites using a uniform co-precipitation method combined with thermal treatment [34]. Hu et al. employed hydrothermal and physical vapor deposition methods for similar purposes [35]. However, these methods involve complex multi-step processes that complicate the factors affecting device performance, making detailed quantitative analysis challenging. In contrast, our research presents a simpler, cost-effective method for preparing TiO_2_–α-Ga_2_O_3_ heterostructures through facile oxidation and hydrothermal reaction techniques. This method is used to fabricate UVC/UVA broad-band photodetectors, allowing for independent detection of UVA or UVC. The potential mechanisms underlying the broad-band detection capabilities of these PDs will also be examined. Additionally, the self-powered photoresponse characteristics based on the interfacial contact and photovoltaic properties of the TiO_2_–α-Ga_2_O_3_ are discussed. An optical encryption communication system for secure data transmission was established, utilizing UVC as the information carrier and UVA for key transmission [36,37]. This study provides a viable approach to preparing TiO_2_–α-Ga_2_O_3_ heterostructures and offers a simple, practicable, and feasible method for fabricating self-powered UVC/UVA broad-band PDs. These attributes address the increasing demand for multifunctional UV PDs and pave the way for broader applications in secure communication, germicidal disinfection, missile interception, and physical protection.

## 2. Experimental Process

### 2.1. Material Preparation

**Figure 1:**
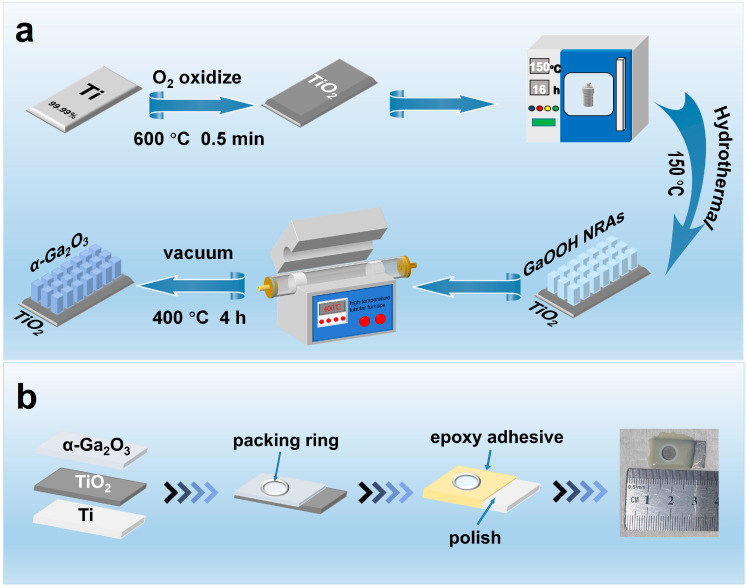
(**a**) Flow chart of TiO_2_–α-Ga_2_O_3_ preparation. (**b**) Schematic diagram of encapsulation procedure.

This experiment used TiO_2_ as the substrate and prepared a precursor solution with a pH of 5. At a temperature of 150 °C, α-Ga_2_O_3_ was grown in TiO_2_ by the hydrothermal method, as shown in Figure 1a [38]. Hydrothermal synthesis is a wet chemical method that simplifies the fabrication process compared to complex multi-step techniques such as chemical vapor deposition, molecular beam epitaxy, or sputtering. Its relatively straightforward process, combined with annealing, helps lower production costs and enhance efficiency. First, titanium sheets (purity ≥ 99.99%, thickness 1 mm) were cut to 1.2 × 2.2 cm^2^ and successively cleaned with anhydrous ethanol, acetone, and deionized (DI) water. Then, the titanium sheets were dried and oxidized in a tube furnace at 600 °C for half a minute to form a thin layer of TiO_2_ onto the surface of titanium sheets [39]. The TiO_2_ was used as the substrate for the growth of GaOOH nanorods (NRs) and the lower titanium sheet functioned as a conductor layer. The precursor solution consisted of 0.39 M Ga(NO_3_)_3_ (gallium(III) nitrate hydrate 99.9% purity) and 0.1 M sodium hydroxide (NaOH) [40]. The oxidized titanium sheet was then placed in a 50 mL Teflon-lined stainless-steel autoclave containing the precursor solution. After the autoclave reached a temperature of 150 °C and was maintained for varying durations, specifically, 12, 14, 16, and 18 h, it was allowed to naturally cool to room temperature. Subsequently, the samples were thoroughly washed several times and were annealed at 400 °C for 4 h. In this manner, TiO_2_–α-Ga_2_O_3_ composites were synthesized. Figure 1b displays the scheme of the preparation process of the photoelectrode. The TiO_2_–α-Ga_2_O_3_ was placed on a clean microscope slide, and the tetrafluoroethylene ring (diameter: 6 mm) was fixed to the sample. The device was coated with an epoxy AB glue and the photoelectrode was finally obtained after vacuum hardening. To assess the growth of α-Ga_2_O_3_, a portion of pure TiO_2_ that had undergone oxidation was subjected to the same encapsulation process.

During the experiment, we identified and addressed several potential sources of error. Firstly, to ensure the oxidation of the Ti sheets occurred in a pure oxygen environment, we performed three purges to avoid introducing impurities. Secondly, the concentration and pH of the precursor solution could affect the quality and distribution of Ga_2_O_3_ growth. To maintain consistency, we accurately weighed and prepared the solution for each experiment, thoroughly mixed it using a magnetic stirrer, and precisely controlled the pH with a pH meter and pipette. The airtightness, temperature, and duration of the hydrothermal reaction were also crucial. We used a bench vise to ensure the autoclave was tightly sealed, strictly controlled the sterilizer’s temperature, and employed a high-precision timer to monitor reaction time. Despite these measures, temperature fluctuations and timing deviations due to equipment limitations and external environmental changes remain potential areas for improvement.

### 2.2. Characterization

The microstructure and elemental composition were characterized using scanning electron microscopy (SEM, FEI Inspect F50, Hillsboro, OR, USA) and energy-dispersive spectrometry (EDS). Chemical bonding states were analyzed by X-ray diffraction (XRD, Bruker D8 ADVANCE A25X (Billerica, MA, USA), Cu Ka1 radiation: λ = 0.1540598 nm) and X-ray photoelectron spectroscopy (XPS, Thermo Scientific, Waltham, MA, USA, Escalab 250xi). Transmission electron microscopy (TEM) was employed to further characterize the microstructure and chemical composition of the materials. Electrochemical properties were evaluated using a three-electrode system, with the TiO_2_–α-Ga_2_O_3_ sample serving as the working electrode, with an effective area of 0.2826 cm^2^. A saturated calomel electrode served as the reference electrode to provide a stable reference potential, while a platinum metal sheet acted as the auxiliary electrode to complete the polarized circuit. The electrolyte used a 0.1 M anhydrous sodium sulfate solution. Among them, the UV (254 nm) light source for testing samples is provided by the UVLS-28 EL UV lamp (AnalytikJena AG, Jena, Germany) and the UV (325 nm) light source is provided by the UV325-01-BL UV lamp (Xiaoxiao Photon Technology Co., Ltd., Shanghai, China).

## 3. Results and Discussion

**Figure 2:**
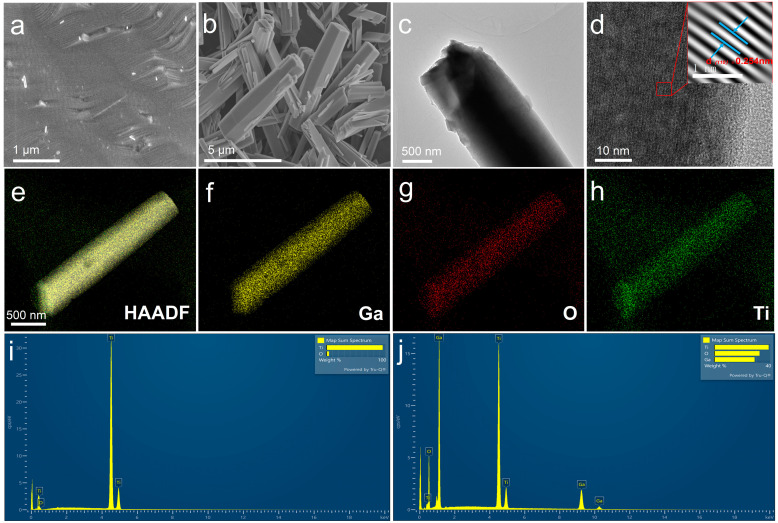
Crystal structure properties of TiO_2_/TiO_2_–α-Ga_2_O_3_ NRs. (**a**) Top-view SEM image of TiO_2_. (**b**–**d**) SEM/TEM images of TiO_2_–α-Ga_2_O_3_ composites at different magnifications. (**e**–**h**) HADDF of TiO_2_–α-Ga_2_O_3_ and mapping of corresponding elements (Ga, O, and Ti). (**i**) EDS spectra of TiO_2_. (**j**) EDS spectra of TiO_2_–α-Ga_2_O_3_.

In this study, we utilized the hydrothermal method to synthesize a TiO_2_–α-Ga_2_O_3_ composite as the photosensitive material for self-powered UVC/UVA broad-band PDs. SEM and TEM characterization were conducted on both TiO_2_ and TiO_2_–α-Ga_2_O_3_ to examine their structural morphology. Figure 2a depicts the rough surface of TiO_2_ at the microscopic level, facilitating efficient attachment of GaOOH NRs. Figure 2b presents a high-magnification SEM image of TiO_2_–α-Ga_2_O_3_, which reveals the uniform growth of α-Ga_2_O_3_ NRs on the TiO_2_ substrate. The nanorods’ tips are arranged in a diamond-like pattern, with a relatively uniform morphology and size distribution, although the surface appears rough and small particles are less ordered. A more detailed microscopic image of the NR in Figure 2c confirms that the fabricated α-Ga_2_O_3_ has a well-defined rod structure. Figure 2d displays a high-resolution TEM image of α-Ga_2_O_3_ that possesses a lattice spacing of 0.254 nm. This corresponds to the (110) crystal plane of α-Ga_2_O_3_ (JADE#43-1013). Zhang et al. synthesized α-Ga_2_O_3_ nanoribbons with a lattice spacing of 0.254 nm, corresponding to the (110) crystal plane of α-Ga_2_O_3_, consistent with our findings [41]. Figure 2e presents a high-angle annular dark-field (HAADF) TEM image of the TiO_2_–α-Ga_2_O_3_, accompanied by elemental mapping of Ga (Figure 2f), O (Figure 2g), and Ti (Figure 2h). The results show that Ga and O elements are uniformly distributed on the single NR, while the distribution of Ti is more extensive, confirming the successful growth of α-Ga_2_O_3_ NRs on TiO_2_ substrates [42]. In Figure 2h, it is clearly observed that Ti elements are distributed on the Ga_2_O_3_ NRs, which is due to a small amount of Ti elements overflowing from the TiO_2_ substrate and doped into the Ga_2_O_3_ NRs during the hydrothermal synthesis process. In Figure 2i, the EDS analysis of the TiO_2_ sample reveals the presence of Ti and O elements, indicating successful oxidation of the titanium sheets. In Figure 2j, the EDS analysis of the TiO_2_–α-Ga_2_O_3_ sample additionally identifies the presence of Ga elements, which confirms the successful growth of α-Ga_2_O_3_ on the TiO_2_ substrate. The chemical composition of the TiO_2_–α-Ga_2_O_3_ heterostructure includes Ga, O, and Ti elements, as further evidenced by these results.

**Figure 3:**
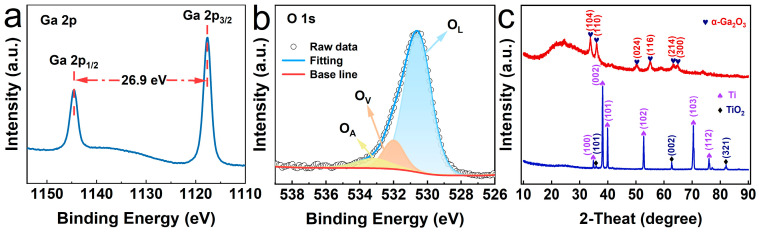
XPS spectra of (**a**) Ga and (**b**) O elements. (**c**) XRD spectra.

To further investigate the structural characteristics of TiO_2_–α-Ga_2_O_3_, Figure 3a reveals the core-level spectrum of Ga 2p, featuring two peaks at 1143.9 eV and 1117.0 eV, which correspond to the binding energies of Ga 2p_1/2_ and Ga 2p_3/2_, respectively. The peak separation is approximately 26.9 eV and the intensity ratio is 1:2. Ni et al. synthesized N-doped α-Ga_2_O_3_@C@G composites as an anode for lithium-ion batteries, in which the binding energies of Ga 2p_1/2_ and Ga 2p_3/2_ are 1145.7 eV and 1118.8 eV, respectively. This is in good agreement with our results [43]. For the O 1s core-level spectrum (as shown in Figure 3b), three peaks can be observed: 530.6 eV (O_L_), 532.0 eV (O_V_), and 533.3 eV (O_A_). The O_L_ peak signifies lattice oxygen, corresponding to the overlapping contributions of O-Ga and O-Ti bonds in TiO_2_–α-Ga_2_O_3_ [44,45]. The O_V_ peak represents vacancy oxygen, indicating a high density of oxygen vacancies in TiO_2_–α-Ga_2_O_3_. The O_A_ peak corresponds to the adsorbed oxygen, suggesting chemisorbed oxygen on the α-Ga_2_O_3_ surface, which is mainly attributed to the presence of organic compounds associated with amorphous carbon [46,47]. XRD spectra of TiO_2_ and TiO_2_–α-Ga_2_O_3_ are provided in Figure 3c. An oxide layer is formed on the Ti substrate at 600 °C. Diffraction peaks at 35.9°, 62.8°, and 82.1° correspond to the (101), (002), and (321) planes of TiO_2_ (JADE#21-1276), respectively, matching well with the rutile phase TiO_2_. Six distinct 2θ peaks at 33.7°, 35.9°, 50.2°, 55.0°, 63.2°, and 65.0° correspond to the (104), (110), (024), (116), (214), and (300) planes of α-Ga_2_O_3_ (JADE#43-1013), respectively, demonstrating the smooth growth of α-Ga_2_O_3_ via hydrothermal and annealing methods. Notably, the peak corresponding to the (104) plane is the most intense, suggesting preferential growth of α-Ga_2_O_3_ along this direction [48]. He et al. reported the synthesis of α-Ga_2_O_3_/Cu_2_O p-n junction and its self-powered feature. The XRD peaks of this heterostructure at 36.0° and 64.7° correspond to the (110) and (300) crystal planes of α-Ga_2_O_3_, respectively, which are in good agreement with our work [49].

**Figure 4:**
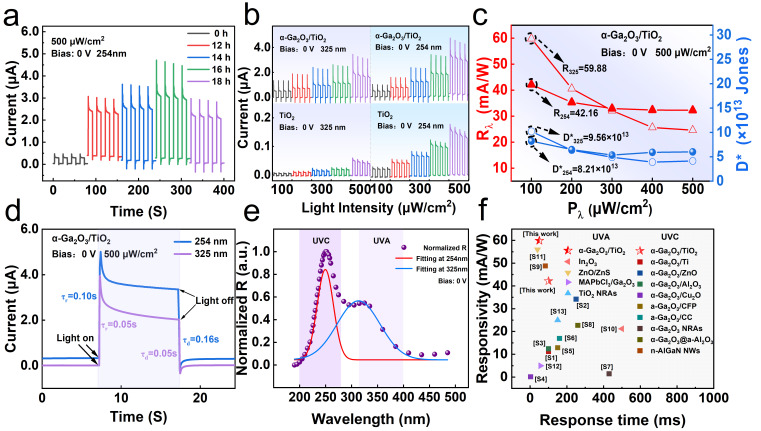
Photoresponse characteristics of TiO_2_ and TiO_2_–α-Ga_2_O_3_ photoelectrochemical (PEC)-type PDs. (**a**) I-t response of five TiO_2_–α-Ga_2_O_3_ samples with different hydrothermal times under UV irradiation at 254 nm. (**b**) I-t curves of TiO_2_ and TiO_2_–α-Ga_2_O_3_ (12, 14, 16, and 18 h) under 254/325 nm with different light intensities irradiation. (**c**) R and D* of the TiO_2_–α-Ga_2_O_3_ PD. (**d**) Response/recovery times at 500 mW/cm^2^ and the wavelengths of 254/325 nm. (**e**) Spectral response of the TiO_2_–α-Ga_2_O_3_ PEC UV PD at 0 V. (**f**) Comparison of the characterization parameters of reported works.

To fabricate high-performance PDs, we chose to composite α-Ga_2_O_3_ on TiO_2_ sheets using a hydrothermal method. By varying the hydrothermal reaction time (12, 14, 16, and 18 h), α-Ga_2_O_3_ NRs were synthesized to construct different TiO_2_–α-Ga_2_O_3_ samples [50]. Figure 4a shows the I-t response curve of the TiO_2_–α-Ga_2_O_3_ PD at 254 nm and 0 V bias for different hydrothermal times. It reveals that all five PDs have self-powered characteristics and photocurrents show good stability and repeatability. Clearly, the photocurrents of four samples are all higher than that of pristine sample (0.3 μA) and the photocurrent of sample 16 h reaches as high as 3.0 μA. The PDs exhibit a gradient variation corresponding to the growth time. When the hydrothermal growth time deviates from the optimal reaction time of 16 h, either shortening or lengthening this duration does not contribute to performance improvement. Thus, extending the hydrothermal growth time improves the material’s crystallinity; however, excessively long growth times may cause the nanorods to detach from the substrate. Consequently, subsequent work will be conducted using PEC-type UV PD based on this optimal reaction time. The initial sharp peaks observed in the I-t curves reflect the rapid accumulation and release of charge in the circuit, a crucial feature of self-powered photodetectors. This rapid response capability is essential for high-speed photonic signal conversion. When developing new materials, stability and repeatability are crucial attributes that directly affect the reliability and efficiency of materials. To evaluate the stability of TiO_2_–α-Ga_2_O_3_, the sample was subjected to a light intensity of 0.5 mA cm^−2^ while maintaining a voltage bias of 0 V, as illustrated in Figure S1. The current density trend was observed by monitoring the current change over a period of 400 s. The TiO_2_–α-Ga_2_O_3_ demonstrated excellent stability throughout this process. Even over prolonged exposure to light, the photocurrent density was maintained at a high level, which not only demonstrates the material’s stability but also indicates its excellent cyclic performance. Figure 4b shows the relationship between bright/dark currents and optical power densities of TiO_2_ and TiO_2_–α-Ga_2_O_3_ under UV light (325/254 nm) and 0 V bias. The TiO_2_–α-Ga_2_O_3_ exhibits a photocurrent of 0.5 μA at 100 μW/cm^2^ under 325 nm UV light, which increases to 1.4 μA at 500 μW/cm^2^. Under 254 nm UV light, the photocurrent is 0.5 μA at 100 μW/cm^2^ and it rises to 3.0 μA at 500 μW/cm^2^. Compared to pristine TiO_2_, TiO_2_–α-Ga_2_O_3_ exhibits a significantly enhanced response under 254 nm and 325 nm UV illuminations, demonstrating excellent detection characteristics at both wavelengths. These results indicate that the photocurrent in TiO_2_–α-Ga_2_O_3_ is closely related to light intensity. Additionally, the R is an important index for photodetectors [51]. The D* is a significant parameter related to noise equivalent power, which can be used to evaluate the ability of devices to detect weak signals in noisy environments [52]. These parameters can be expressed as:

(1) $$R=\frac{I_{L}-I_{D}}{PS}$$

(2) $$D^{*}=\frac{R\sqrt{S}}{\sqrt{2eI_{D}}}$$

where I_L_, I_D_, P, S, and e are the photocurrent, dark current, optical power density, effective light area, and electron charge, respectively. When an external light signal illuminates the device, photons with energies surpassing the bandgap of the photosensitive material excite electrons from the valence band to the conduction band; then, nonequilibrium charge carriers are generated. These carriers, upon being collected by the electrodes, enable external circuitry to detect resultant electrical signals, facilitated either by the built-in electric field or an external bias voltage. The R quantitatively characterizes the extent of change in the electrical signal generated by the detector upon exposure to external light. In Figure 4c, the trends of R and D* under 254/325 nm light are illustrated; at an intensity of 100 μW/cm^2^, the R takes the maximum values of 42.16/59.88 mA/W and the D* takes the maximum values of 8.21 × 10^13^/9.56 × 10^13^ Jones, respectively. As incident light increases from 0.1 to 0.5 mW/cm^2^, the R of the self-powered PD decreases from 42.16/59.88 to 32.27/24.59 mA/W and D* decreases from 8.21 × 10^13^/9.56 × 10^13^ to 5.35 × 10^13^/3.66 × 10^13^ Jones, respectively. This indicates that, under intense illumination, a significant number of photogenerated charge carriers are produced, leading to increased carrier recombination rates and decreased photon utilization efficiency, resulting in decreased R and D* [53]. Bansal et al. presented highly efficient p^+^-BLG and nanowires-based single- and dual-junction broad-band photodetectors at 300 K. Specifically, the double-heterojunction device exhibited the highest D* of 4.2 × 10^12^ Jones at a 480 nm wavelength. Notably, the study has achieved D* of 5.35 × 10^13^/3.66 × 10^13^ Jones, which is an improvement over recent work in this field, enabling more effective discrimination of target signals from background noise, demonstrating outstanding signal detection capability [54].

To further characterize the sensitivity of PDs, the response and recovery properties of self-powered PEC-PDs need to be investigated [54]. The rise and decay times (τ_r_/τ_d_) are determined by measuring the time required for the photocurrent increasing from 10% to 90% of its maximum value (τ_r_) and decreasing from 90% to 10% of its maximum value (τ_d_), respectively. Figure 4d depicts an enlarged view of rise and decay edges of the TiO_2_–α-Ga_2_O_3_ PD under 254/325 nm light with an incident intensity of 500 μW/cm^2^. The τ_r_ of TiO_2_–α-Ga_2_O_3_ at 254 nm is 0.10 s, as well as the τ_d_ being 0.16 s. Meanwhile, the τ_r_/τ_d_ at 325 nm are both 0.05 s. Therefore, it can be observed that the self-powered UVC/UVA broad-band PD developed in this study exhibits rapid response characteristics. Figure 4e shows the wavelength-dependent R of the TiO_2_–α-Ga_2_O_3_ ranging from 200 to 450 nm. It demonstrates a significant responsiveness to UVC and UVA bands, with minimal response to visible light. Two R peaks can be observed: 42.16 mA/W at ~254 nm and 59.88 mA/W at ~325 nm, corresponding to the absorption of Ga_2_O_3_ and TiO_2_, respectively. This is consistent with results reported previously [55,56]. The PD exhibits a response that is folds higher for 254 nm illumination compared to 325 nm, indicating a predominance in UVC detection capability. In this TiO_2_–α-Ga_2_O_3_-based PD, both TiO_2_ and Ga_2_O_3_ layers can function as absorption layers. The TiO_2_ film primarily acts as the UVA light absorption layer due to its shorter bandgap and high R in the UVA band, while Ga_2_O_3_ serves as the UVC absorption layer. This feature makes this PD realize both UVA and UVC detection. Dixit et al. achieved a dual-band UV PD based on ZnO–ZnCr_2_O_4_ nanostructures through a simple solution process [57]. The device has dominant responses in UVC (210 nm) and UVA (350 nm) regions. Notably, the τ_r_/τ_d_ of the transient response were determined to be approximately 21 s and 45 s under 350 nm excitation, respectively. Also, the τ_r_/τ_d_ were measured to be approximately 30 s and 40 s under 250 nm excitation, respectively. The response time in this study is significantly faster than that of the ZnO–ZnCr_2_O_4_, indicating outstanding performance in broad-band detection. In brief, this PD demonstrates an intriguing broad-band detection capability for both UVC and UVA radiation.

Figure 4f provides a concise comparison of the R and τ_r_ between the self-powered PEC photodetector developed in this study and previously reported UV photodetectors. Notably, our device demonstrates superior overall performance compared to other PEC UV photodetectors. For instance, Liu et al. prepared self-powered solar-blind DUV PD based on α-Ga_2_O_3_ NRs/ZnO by hydrothermal and post-annealing methods and achieved a high performance of 34.2 mA/W [58]. However, its response time is as long as 250 ms, which limits its practical application. Zhang et al. deposited 3D-structured amorphous Ga_2_O_3_ films on carbon cloth (CC) using room-temperature magnetron sputtering. The PD based on a-Ga_2_O_3_/CC composite demonstrates excellent flexibility and stability, while its responsivity was relatively low (16.98 mA/W) [59]. As such, this work has made significant progress in improving responsiveness and efficiency, clearly demonstrating its potential and advantages in practical applications. In contrast, Wang et al. fabricated self-powered α-Ga_2_O_3_/Cu_2_O heterojunction PDs with solar-blind UV-visible photodetection. In the absence of bias, the device demonstrated a responsivity of 0.12 mA/W at 254 nm, with τ_r_/τ_d_ of 2.48/11.72 ms, respectively [60]. At a 475 nm incident light, its responsivity reached 19 mA/W and τ_r_/τ_d_ were 0.96/9.12 ms, respectively. This work is in line with their broad-band design concept; however, compared to α-Ga_2_O_3_/Cu_2_O, the response range in this work is controlled in UVC and UVA, which makes it independent of the visible region and will be more resistant to solar interference. In short, the self-powered TiO_2_–α-Ga_2_O_3_ heterojunction PDs prepared in this work have high responsivity, fast response, and broad-band detection characteristics, as well as the advantages of ease of preparation, low cost, and high performance, which make them potentially useful for optical communication.

**Figure 5:**
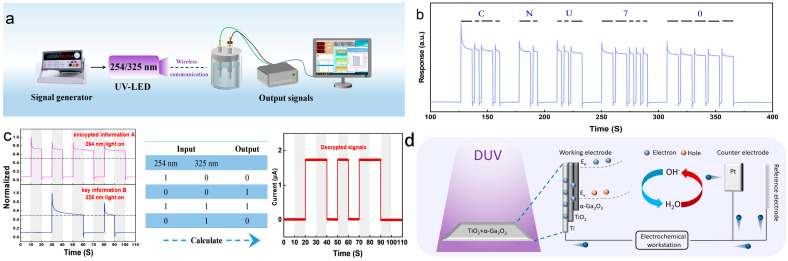
(**a**) Schematic diagram of the UV optical communication setup utilizing the self-powered TiO_2_–α-Ga_2_O_3_ PEC-PD. (**b**) Morse-code-encoded digital data waveforms received by the PD. (**c**) Schematic diagram of the calculation process of broad-band secure optical communication. (**d**) Schematic diagram of self-powered TiO_2_–α-Ga_2_O_3_ for PEC testing under UV irradiation.

Given the exceptional PEC response performance and self-powered capabilities demonstrated by the TiO_2_–α-Ga_2_O_3_ material, we extended our research to explore its potential applications in the domain of safe optical communication. We developed an innovative system utilizing a PEC-PD as a self-powered optical signal receiver. This system is integrated with a programmable digital power supply for the optical signal generator and a 254 nm UV LED lamp, creating a highly efficient and stable communication platform, as depicted in Figure S2. Figure 5a illustrates the schematic of the measurement system. It demonstrates the transmission of international Morse code using the PEC-PD [61]. In Morse code, each letter is represented by a series of dots and dashes, as shown in Figure S3. Initially, we used a programmable digital power supply to precisely control the voltage output amplitude and duration, thereby finely tuning the brightness and duration of the UV signals. This allowed us to encode the information sequences “CNU” and “70” into Morse code patterns of dots and dashes. These UV signals were then wirelessly transmitted in open space and detected by the TiO_2_–α-Ga_2_O_3_ PD, which further converted into electrical signals, as depicted in Figure 5b. The electrical signal waveform displayed a sequence of three communication states: dot, dash, and silence. By decoding these sequences according to Morse code, we were able to accurately decipher the letters “CNU” and the digits “70”. Furthermore, the photodetector exhibited good stability and high responsiveness throughout the process. These findings conclusively demonstrate the feasibility and reliability of TiO_2_–α-Ga_2_O_3_ PD in constructing high-speed, low-power, and interference-resistant UV light communication systems.

With the rapid advancements in optical communication, the frequent transmission of data in open space increases the risk of interception, thereby demanding enhanced security measures. In this study, we harness the broad-band light response of TiO_2_–α-Ga_2_O_3_ PD to propose a more secure and confidential communication approach. Our communication platform employs a similar approach, substituting the light source with UVC and UVA, while still using a programmable digital power supply for information sequence encoding. Specifically, UVC at 254 nm is utilized to transmit encrypted information A, while UVA at 325 nm conveys the corresponding binary-coded key information B. At the PEC-PD receiver, a standardized current normalization process is implemented, wherein photocurrents above 0.5 are decoded as binary “1” and dark currents below 0.5 as binary “0”. The encryption protocol is defined as follows: when a binary bit in B is “0”, the corresponding information in A (either “0” or “1”) is regarded as false and its inverse (“1” or “0”) is output. Conversely, when a binary bit in B is “1”, the corresponding information in A remains unchanged, with “0” or “1” being output. For instance, an encrypted message A using UVC as “101011011” and a binary key message B from UVA as “001110010” are decoded through the protocol to yield the actual message “011010110”. The computational process for secure optical communication is illustrated in Figure 5c. The improved design significantly enhances security while ensuring excellent performance. It incorporates a dual-layer protection mechanism, ensuring that only a photodetector with UVC/UVA light response and the agreed encryption algorithm can retrieve the real information. These findings conclusively highlight the significant potential of TiO_2_–α-Ga_2_O_3_ in secure communication and efficient data transmission, establishing a strong foundation for its widespread application in practical scenarios. The UVA/UVC dual-band detector, as an advanced sensor technology, is capable of accurately detecting radiation in both UVA and UVC wavelengths. This capability opens up broad application prospects across various fields, including science, industry, and environmental monitoring.

In brief, as shown in Figure 5d, the self-powered characteristics of the TiO_2_–α-Ga_2_O_3_ heterostructure arise from two key factors: firstly, the inherent electric field effectively suppresses rapid carrier recombination at the interface; secondly, photoexcited electrons and holes follow distinct transport pathways, creating a current loop through external connections. Specifically, under 254/325 nm illumination, photo-generated holes in α-Ga_2_O_3_ oxidize hydroxyl anions (OH^−^) in the solution to hydroxyl radicals (OH*) at the solid–liquid interface due to the inherent electric field. Continuous UVC/UVA light irradiation of the TiO_2_–α-Ga_2_O_3_ heterostructure enables the realization of self-powered characteristics without an applied bias. Beyond the optical communication domain discussed in this paper, this technology holds potential value in other areas. For example, in environmental monitoring, UVA/UVC detectors can be used for real-time air and water quality assessment, evaluating the impact of UV radiation on environmental organisms, and thereby protecting ecosystems and public health. In medical diagnostics, such detectors could aid in detecting diseases such as skin cancer by analyzing skin tissue responses to different UV wavelengths. In safety systems, UVA/UVC detectors can monitor the radiation levels of UVC disinfection lamps to ensure the effectiveness of the disinfection process and safeguard the safety of personnel. Due to their high sensitivity and responsiveness, these dual-band detectors can detect minute changes in radiation with precision and timeliness, which is crucial for ensuring the accuracy of monitoring results and the safety of practical applications.

## 4. Conclusions

In summary, TiO_2_–α-Ga_2_O_3_ was prepared through facile oxidation, followed by the hydrothermal reaction, as supported by different approaches of characterization. The fabricated bilayer structure demonstrated in this study features self-powered UVC/UVA broad-band detection capability, in which the optimal response was observed at the growth time of 16 h. This PD exhibited two response peaks, ~254 (UVC) and 325 nm (UVA), and displayed the possibility of broad-band detection. Owing to the shorter bandgap and high R in the UVA band, the TiO_2_ bottom layer can efficiently absorb UVA photons, while Ga_2_O_3_ contributes to the UVC-dominated detection capability. The PD exhibits promising R (42.16/59.88 mA/W) and D* (8.21 × 10^13^/9.56 × 10^13^ Jones), indicating a practicable photo-detectivity in two bands. A secure optical communication system utilizing UVC and UVA wavelengths is proposed, leveraging its broad-band response characteristics to establish two independent optical channels, in which UVC serves as the information carrier and UVA as the key. This conceptual design holds promising applications in communication, where secure and reliable information can be transmitted using encryption algorithms. Further research is needed to optimize the performance of these broad-band diodes, such as enhancing detection capabilities for UVC and UVA by controlling different bias voltages, which will aid in precise wavelength selection and signal processing, thereby improving overall communication efficiency and security. Overall, this study not only provides crucial scientific insights into TiO_2_–α-Ga_2_O_3_-based UVC/UVA detection but also paves the way for developing multifunctional, high-performance multi-band photodetectors. These findings are significant for both scientific research and industrial applications, particularly in secure communications and environmental monitoring.

## Data Availability

The raw data supporting the conclusions of this article will be made available by the authors on request.

## Supplementary Materials

The following supporting information can be downloaded at: https://www.mdpi.com/article/10.3390/ma17164103/s1, Figure S1: Long-time stability tests. Figure S2: The designed system consists of a PEC-PD as the self-powered optical signal receiver and an optical signal generator with a programmable digital power supply and 254/325 nm UV LED lights. Figure S3: The letters and numbers selected in the box above are those that appeared in the experiment. References [58,59,60,62,63,64,65,66,67,68,69] are cited in the Supplementary Materials.
