# Supplementary material for: A Facile Synthesis of TiO2–α-Ga2O3-Based Self-Powered Broad-Band UVC/UVA Photodetector and Optical Communication Study
Source: Materials (Basel). 2024 Aug 19;17(16):4103. doi: 10.3390/ma17164103 (PMC11356172; doi:10.3390/ma17164103)
Supplement: Supplementary file 1 [file materials-17-04103-s001.zip › materials-3139121-supplementary.pdf]

A Facile Synthesis of TiO_2–α-Ga_2O_3-Based Self-Powered Broad-Band UVC/UVA Photodetector and Optical Communication Study

Wenxing Zhang [†], **Anqi Xu** [*,†], **Xin Zhou, Dan Zhang and Honglin Li** [*]

Chongqing Key Laboratory of Photo-Electric Functional Materials, College of Physics and Electronic Engineering, Chongqing Normal University, Chongqing 401331, China
* 18678613265@163.com (A.X.); lin@cqnu.edu.cn (H.L)
† These authors contributed equally to this work.

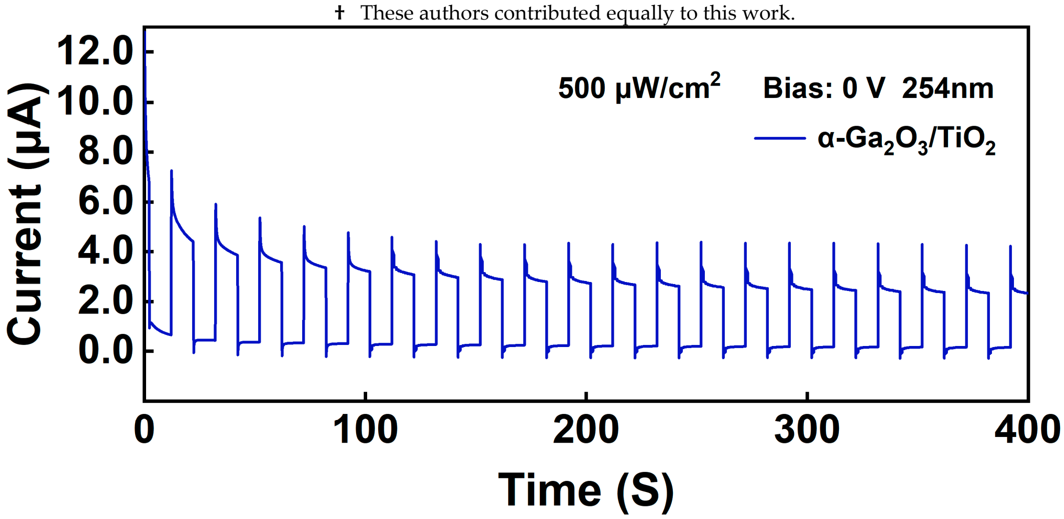

Figure S1 Long-time stability tests.

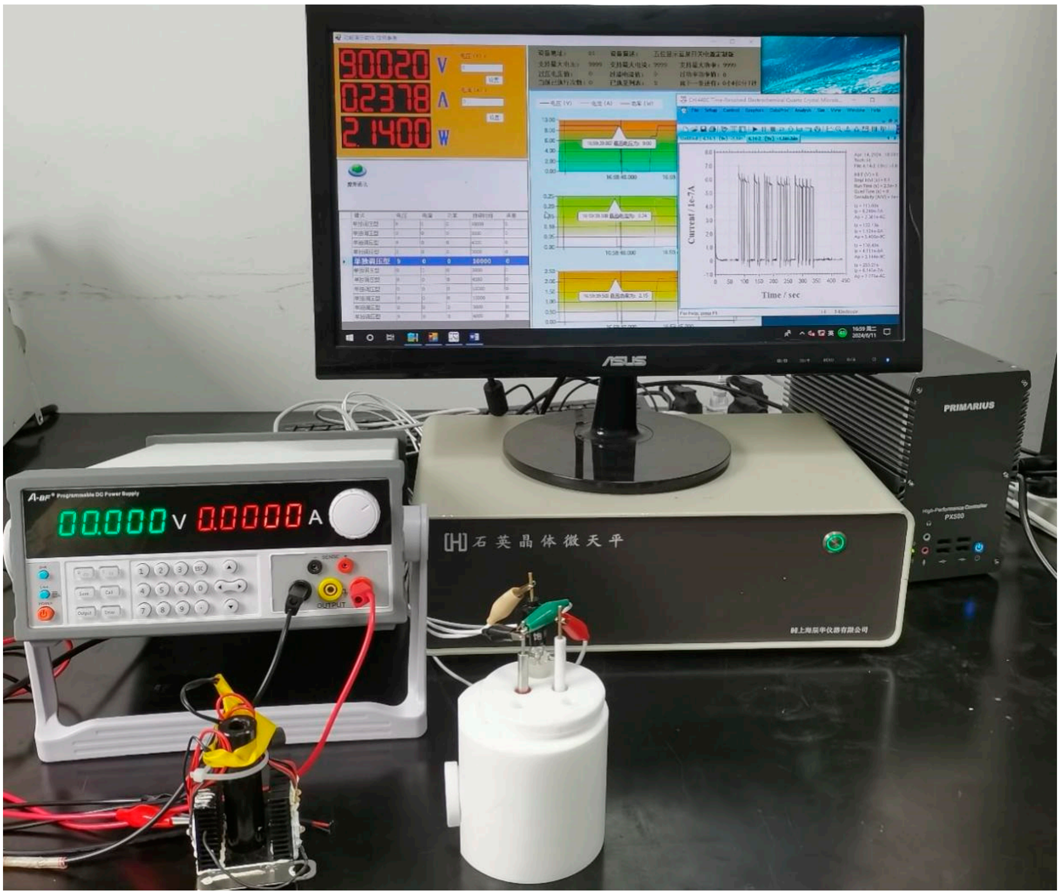

Figure S2 The designed system consists of a PEC-PD as the self-powered optical signal receiver and an optical signal generator with a programmable digital power supply and 254/325 nm UV LED lights.

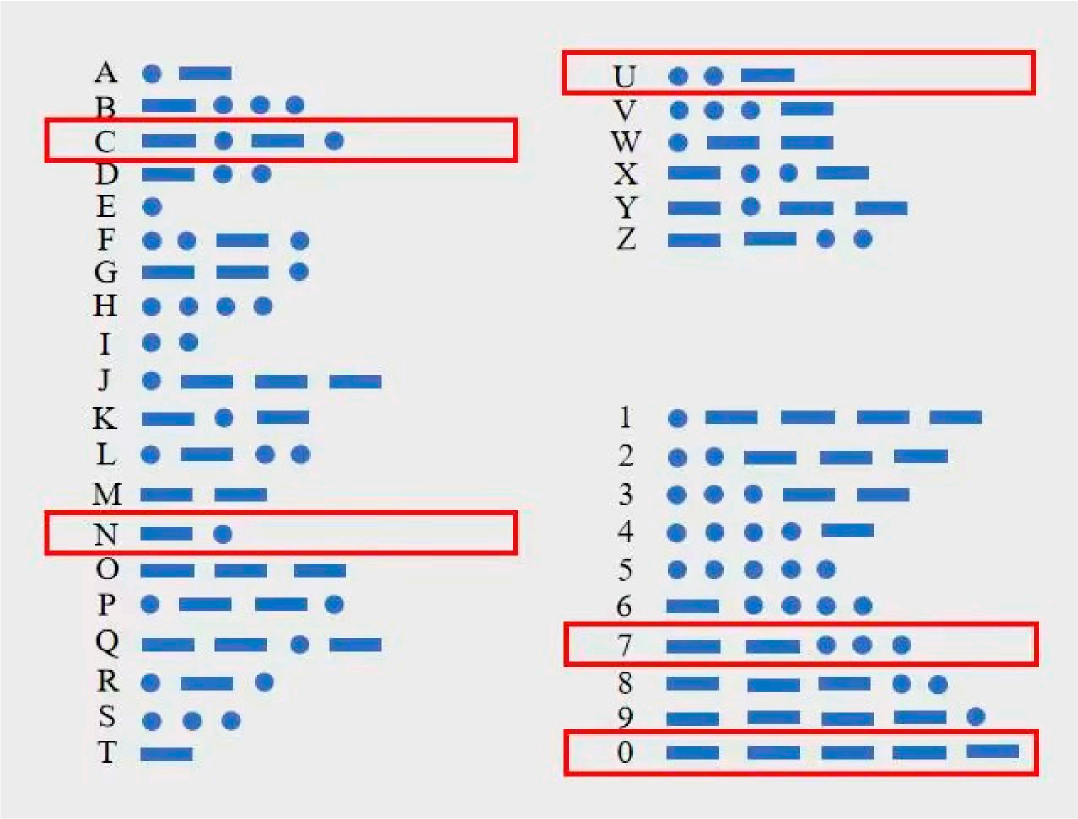

Figure S3 The letters and numbers selected in the box above are those that appeared in the experiment.

References

1. G. Wang, et al., "A wire-shaped photoanode of the solar-blind photoelectrochemical-type photodetector based on an α-Ga_2O_3 nanorods/electrolyte solid/liquid heterojunction," Applied Physics Letters **122**(2023).
2. W. Liu, et al., "Construction of α-Ga_2O_3-ZnO heterojunction for a promoted performance applied in self-powered solar blind photodetector," The European Physical Journal Applied Physics **97**, 57 (2022).
3. J. Zhang, et al., "Nano tree-like branched structure with α-Ga_2O_3 covered by γ-Al_2O_3 for highly efficient detection of solar-blind ultraviolet light using self-powered photoelectrochemical method," Applied Surface Science **541**, 148380 (2021).
4. X. Wang, et al., "Self-powered solar-blind ultraviolet-visible Cu_2O/Ga_2O_3 photodetectors," Journal of Materials Chemistry C **12**, 8944-8951 (2024).
5. L. Huang, et al., "A simple, repeatable and highly stable self-powered solar-blind photoelectrochemical-type photodetector using amorphous Ga_2O_3 films grown on 3D carbon fiber paper," Journal of Materials Chemistry C **9**, 10354-10360 (2021).
6. D. Zhang, et al., "Flexible self-powered solar-blind UV photodetectors based on amorphous Ga_2O_3 modified carbon fiber cloth," Journal of Alloys and Compounds **969**, 172483 (2023).
7. K. Chen, et al., "Photoelectrochemical self-powered solar-blind photodetectors based on Ga_2O_3 nanorod array/electrolyte solid/liquid heterojunctions with a large separation interface of photogenerated carriers," ACS Applied Nano Materials **2**, 6169-6177 (2019).
8. Wang X, Ding K, Huang L, et al. Enhancing the performance of Self-Powered Deep-Ultraviolet photoelectrochemical photodetectors by constructing α-Ga2O3@ a-Al2O3 Core-Shell nanorod arrays for Solar-Blind imaging[J]. Applied Surface Science, 2024, 648: 159022.

9. D. Wang, et al., "Highly uniform, self-assembled AlGaN nanowires for self-powered solar-blind photodetector with fast-response speed and high responsivity," Advanced Optical Materials **9**, 2000893 (2021).
10. M. Cui, et al., "MOF-derived In_2O_3 microrods for high-performance photoelectrochemical ultraviolet photodetectors," ACS Applied Materials & Interfaces **14**, 39046-39052 (2022).
11. H. Lin, et al., "High-performance self-powered photodetectors based on ZnO/ZnS core-shell nanorod arrays," Nanoscale research letters **11**, 1-7 (2016).
12. S. Liu, et al., "Polycrystalline perovskite $CH_3NH_3PbCl_3$/amorphous Ga_2O_3 hybrid structure for high-speed, low-dark current and self-powered UVA photodetector," Journal of Alloys and Compounds **890**, 161827 (2022).
13. Xie Y, Wei L, Wei G, et al. A self-powered UV photodetector based on TiO_2 nanorod arrays[J]. Nanoscale research letters, 2013, 8: 1-6.
